# Prognostic Implications of Quantifying Haemodynamic Support: Looking Beyond a Snapshot Score

**DOI:** 10.21470/1678-9741-2021-0106

**Published:** 2022

**Authors:** Rohan Magoon, Jes Jose

**Affiliations:** 1 Department of Cardiac Anaesthesia, Atal Bihari Vajpayee Institute of Medical Sciences (ABVIMS) and Dr. Ram Manohar Lohia Hospital, Baba Kharak Singh Marg, New Delhi, India. E-mail:; 2 Department of Cardiac Anaesthesia, Sri Jayadeva Institute of Cardiovascular Sciences and Research, Jayanagar, Bengaluru, Karnataka, India

Dear Editor,

Baysal et al.^[1]^ study, published recently in the Brazilian Journal of Cardiovascular Surgery, epitomizes an important concept of objectively quantifying the degree of haemodynamic support by computing the vasoactive-inotropic score (VIS). The authors attribute prognostic implications to a postoperative VIS >5.5 as an independent predictor of morbidity and mortality after coronary artery bypass grafting in their prospective evaluation.

However, the authors’ findings need to be carefully interpreted in the absence of VIS estimation in the intensive care unit (ICU) in the index study, particularly when the predictive links of postoperative VIS are being sought with mortality and dynamic ICU morbid outcomes, such as renal failure, central nervous damage, etc. in a small cohort of 290 patients^[1]^. Appropriate to the context, Koponen et al.^[2]^ study deserves a mention here which aimed to retrospectively evaluate the association between the highest VIS in the first 24 hours post-ICU admission and a composite poor outcome in 3,213 adult cardiac surgical patients. While their elucidation of a linear increase in the odds of adverse primary postoperative outcome with escalating ICU-VIS scores is noteworthy, it also does adequately emphasize the relevance of a continuous assessment of the VIS scores extending well into the period beyond ICU admission^[2]^.

At the same time, with the understanding of the fact that VIS only allows for the haemodynamic support quantification at a single time-point, the conceptualization of a VIS index by Crow et al.^[3]^ aims at an additional account for the prolonged haemodynamic support requirement^[4]^. They describe a cumulative VIS calculation as follows: VIS_0-24h_(maximum) + VIS_24-48h_(maximum) + 2×VIS_48-72h_(maximum), which is subsequently divided by 10 to compute an integer VIS index. Alongside a heightened discriminative performance when compared to the VIS (maximum) alone, a VIS index ≥3 has been outlined to be associated with an accentuated risk of poor composite outcome after cardiac surgery^[3^,^4]^.

Ahead of the augmented standardization achieved by employing objective haemodynamic support scores like VIS, their outcome predictive potential evaluation needs to be more deliberate with a simultaneous consideration to the two equally critical factors of magnitude and duration of haemodynamic support rather than envisaging a snapshot score concept.
